# Analysis of Comparative Sequence and Genomic Data to Verify Phylogenetic Relationship and Explore a New Subfamily of Bacterial Lipases

**DOI:** 10.1371/journal.pone.0149851

**Published:** 2016-03-02

**Authors:** Malihe Masomian, Raja Noor Zaliha Raja Abd Rahman, Abu Bakar Salleh, Mahiran Basri

**Affiliations:** 1 Enzyme and Microbial Technology Research Centre, Universiti Putra Malaysia, Serdang, Selangor, Malaysia; 2 Faculty of Biotechnology and Biomolecular Science, Universiti Putra Malaysia, Serdang, Selangor, Malaysia; 3 Faculty of Science, Universiti Putra Malaysia, Serdang, Selangor, Malaysia; University of Lincoln, UNITED KINGDOM

## Abstract

Thermostable and organic solvent-tolerant enzymes have significant potential in a wide range of synthetic reactions in industry due to their inherent stability at high temperatures and their ability to endure harsh organic solvents. In this study, a novel gene encoding a true lipase was isolated by construction of a genomic DNA library of thermophilic *Aneurinibacillus thermoaerophilus* strain HZ into *Escherichia coli* plasmid vector. Sequence analysis revealed that HZ lipase had 62% identity to putative lipase from *Bacillus pseudomycoides*. The closely characterized lipases to the HZ lipase gene are from thermostable *Bacillus* and *Geobacillus* lipases belonging to the subfamily I.5 with ≤ 57% identity. The amino acid sequence analysis of HZ lipase determined a conserved pentapeptide containing the active serine, GHSMG and a Ca^2+^-binding motif, GCYGSD in the enzyme. Protein structure modeling showed that HZ lipase consisted of an α/β hydrolase fold and a lid domain. Protein sequence alignment, conserved regions analysis, clustal distance matrix and amino acid composition illustrated differences between HZ lipase and other thermostable lipases. Phylogenetic analysis revealed that this lipase represented a new subfamily of family I of bacterial true lipases, classified as family I.9. The HZ lipase was expressed under promoter *P*_*lac*_ using IPTG and was characterized. The recombinant enzyme showed optimal activity at 65°C and retained ≥ 97% activity after incubation at 50°C for 1h. The HZ lipase was stable in various polar and non-polar organic solvents.

## Introduction

Lipases are glycerol ester hydrolases that catalyze the hydrolysis or synthesis of a broad range of water insoluble esters. They constitute the most important group of biocatalysts for biotechnological application. The ability of lipases to perform very specific chemical transformation (biotransformation) has made them increasingly popular in the food, detergent, cosmetics, organic synthesis, and pharmaceutical industries [1–3].

Lipolytic enzymes have been classified hitherto according to different criteria such as their known substrate specificity, interfacial activation and possessing a movable lid. However, in the last decade, with the discovery of some exceptions, these criteria proved to be unsuitable for classification [4,5]. The increasing knowledge at the atomic level of the 3-D structure of bacterial lipolytic enzymes using either X-ray crystallography or NMR spectroscopy has promoted an attempt to classify these enzymes according to their fold. All lipases as well as the majority of the esterases display the same structural architecture, the so-called α/β hydrolase fold [6]. The growing sequence information of cloned lipases and knowledge of protein 3-D structures has been used to identify possible sequence specific motifs. Lipases share two conserved features, including a serine in a highly conserved GXSXG pentapeptide motif and an aspartate or glutamate residue that is hydrogen bonded to a histidine to form a catalytic triad, and also the oxyanion hole [7–9].

Furthermore, Arpigny and Jaeger [7], by comparing amino acid sequences and the fundamental biological properties of bacterial lipolytic enzymes, classified them into eight families, which are being further extended along with identification of new lipases. The true lipases were ascribed into family I, which was sub-grouped into eight subfamilies [7,8,10]. Among them, *Bacillus* and *Geobacillus* lipases belong to the families I.4 and I.5, which have an alanine residue replacing the first glycine in the conserved pentapeptide: AXSXG [11]. However, the lipases from strains *Bacillus subtilis* and *Bacillus pumilus* stand apart (subfamily I.4) because they are the smallest true lipases known (~20 kDa). Moreover, thermostable lipases from *Bacillus* and *Geobacillus* with more than 90% similarity in their sequences are placed in subfamily I.5. However, they show some differences in their properties such as optimum temperature activity, which has been found between 55 and 70°C. Lipases in family I illustrated a variety of characteristics in activity, thermostability and substrate specificity, and with the discovery of new bacterial lipases, our knowledge of the diversity of lipases will be improved [12].

To date, strain HZ has been the only report of lipase producing bacterium from *Aneurinibacillus* genus [13]. The enzyme reached the highest level of production after 48 h, which is at the end of a stationary phase based on the colony-forming unit (CFU) result. In this work, by using analysis of comparative sequence, genomic data, phylogenetic analysis, homology modeling method and characterization of the recombinant enzyme, we have reported the discovery of a novel lipase from a thermophilic bacterium belonging to an uncharacterized subfamily of family I. Thus, it could be the unique opportunity for a fundamental study on new lipases close to HZ lipase.

## Materials and Methods

### Strains and materials

*Aneurinibacillus thermoaerophilus* strain HZ, which secreted a thermostable and organic solvent-tolerant lipase after 48 h (at the end of a stationary phase), was isolated from a hot spring Sungai Kelah, Malaysia [13,14]. *Escherichia coli* TOP10 was purchased from Novagen. Restriction enzymes and plasmids were purchased from Thermo Scientific. All the chemicals used were of analytical grade.

### Isolation and sequencing of HZ lipase gene

Genomic DNA library method was used to isolate the HZ lipase gene. The genomic DNA was extracted using the DNeasy Blood & Tissue Kit (QIAGEN, Germany) according to the manufacturer’s instructions. In order to prepare a clonable size of genomic DNA, the reaction mixture contained: genomic DNA, 10X reaction buffer, *Eco*RI, and distilled water. The reaction was incubated at 37°C for 1h. Then, it was heat-deactivated by incubating at 65°C for 20 minutes. The DNAs in the range of 2 and 10 kb were excised from the gel and purified using QIAquick Gel Extraction kit (QIAGEN, Germany) according to the manufacturer’s instructions. The pUC19 plasmid was digested using the *Eco*RI restriction enzyme and dephosphorylated by FastAP™ Thermosensitive alkaline phosphatase. The plasmid was then purified using the QIAquick Gel Extraction kit. The purified partially digested genomic DNA was ligated with pUC19 using T4 DNA ligase. The ligation mixture contained: digested plasmid (0.5 μg), partially digested genomic DNA (1 μg), 1.5 μL of 10X ligase buffer, 1 μL of T4 DNA ligase (5U), dH_2_O up to 15 μL. The mixture was incubated at 16°C for 16 h. Transformation of *E*. *coli* TOP10 was performed according to the method described by Sambrook et al. [15]. The transformed cells were transferred to growth medium (SOC), and incubated for 1 h 30 min at 37°C with shaking at 200 rpm. Then transformed cultures were plated onto tributyrin-ampicillin agar, separately and incubated at 37°C. Tributyrin-ampicillin agar was supplemented with Isopropyl-β-D-thiogalactopyranoside (IPTG) (1M, 5 μL) and X-Gal (2% w/v, 50 μL) by spreading on the agar 30 min before transferring transformation culture on the agar. Observation for halo forming white colony was done every 12 to 72 h. Plasmid DNA was extracted by GeneAll^®^ Exprep^TM^ Plasmid Quick Kit (GeneAll, Korea) according to the manufacturer’s instructions.

#### Primer walking

The plasmid of the positive recombinant clone containing an 8 kb insert was extracted and sequenced with universal primers of pUC19 by 1^st^ BASE Laboratories Sdn Bhd (Shah Alam, Selangor, Malaysia). Based on the sequencing result in each step, pairs of primers were designed to amplify the whole insert.

#### Sequencing and analysis of insert gene

DNA sequencing was performed by using Applied Biosystems' highest capacity-based genetic analyzer platforms and the BigDye^®^ Terminator v3.1 cycle sequencing kit chemistry (1^st^ BASE Laboratories Sdn Bhd). The ORF was predicted using the facility at http://www.ncbi.nlm.nih.gov/gorf/gorf.html. Sequence similarity search was conducted with the Basic Local Alignment Search Tool (BLAST) at http://blast.ncbi.nlm.nih.gov/Blast.cgi. Analysis of the insert genes was done by using Biology Workbench (http://www.biology.ncsa.sdsc.edu) and Expasy Molecular Biology Servers (http://www.expasy.org/tools). Lipase sequences for comparative study were retrieved from protein and nucleotide databases on the NCBI Entrez server at http://www.ncbi.nlm.nih.gov/Entrez/. The alignment of the protein sequences was done using GONNET as the protein weight matrix. A combination of 10 and 0.1 were used for gap opening and gap extension penalties, respectively. A phylogenetic tree was constructed by the neighbor-joining method in the Molecular Evolutionary Genetics Analysis 6.06 software (MEGA, version 6.0) [16] under the Poisson correction model of molecular evolution with uniform rate variation among sits or homogeneous patterns among lineages. Alignment gaps were removed from the analysis utilizing the complete deletion option. Bootstrap analyses were conducted with 1000 replicates.

### Nucleotide sequence accession number

The sequence of the HZ lipase gene has been deposited in the GenBank database under accession number GU272057.

### Cloning and expression of the HZ lipase gene

Cloning of the HZ lipase gene (ORF) into pUC19 was performed by incorporating suitable restriction sites at both ends of the gene through amplification, followed by digestion, ligation with the plasmid and transformation. The primers were For PU: 5’-AAG GAG GAA TTC TAT CAT GCA AAA GGA AAG-3’ (*Eco*R1) and Rev PU: 5’-TGC TTC TAG ATT ATC TCA CAG ATA ATG AAC-3’ (*Xba*1) (restriction sites are underlined). Amplification of the HZ lipase gene was performed with gradient PCR. PCR was carried out using *Pfu* DNA polymerase. The positive transformants with halo on tributyrin-ampicillin agar plates were sub-cultured. The existence of the lipase gene (insert) was confirmed by lipase gene amplification (PCR) and sequencing following the plasmid extraction.

To express the HZ lipase gene, cell harboring the recombinant plasmid was propagated in 200 mL of LB-broth supplemented with 50 μg/mL of ampicillin at 37°C with 200 rpm shaking rate. When the culture reached OD_*600nm*_ of 0.5, IPTG (final concentration of 1.0 mM) was added to induce the expression of the cloned lipase gene. The cells were incubated further for 12 h and harvested by centrifugation (10000 ×g, 10 min, 4°C).

### Lipase assay

A ten-milliliter culture was harvested by centrifugation. Then pellet cells were resuspended in 50 mM phosphate buffer pH 7.0, and disrupted by sonication with Branson sonifier 250. After cell disruption, the cell debris was removed by centrifuged at 10000 ×g for 10 min at 4°C. The supernatant was used as recombinant enzyme. Lipase activity was measured by the modified method of Kwon and Rhee [14,17]. Olive oil was used as substrate and 50 mM phosphate buffer pH 7.0 was used to prepare the emulsion and assay mixture. One unit (U) of lipase activity is defined as the rate of 1.0 μmole fatty acid formation per min under standard assay condition.

### Determination of effects of temperature and organic solvents on HZ lipase

To detect the optimal temperature for lipase activity, the assay mixture was equilibrated at the required temperature (at a range of 45–75°C), and 0.05 ml enzyme (0.5 mg/ml) was added to commence the reaction. The impact of temperature on lipase stability was defined by incubating aliquots of HZ lipase for 1 h at different temperatures. Residual activity was analyzed at 65°C.

Various organic solvents were tested at the concentration of 25% (v/v) by adding 0.05 ml of organic solvent to 0.15 ml of HZ lipase (0.5 mg/ml). The mixture was preincubated for 30 min at 50°C under shaking condition (150 rpm) to ensure the continuous mixing of the enzyme and solvent. The stability of enzyme was displayed as the remaining activity assayed relative to the control value. For control, phosphate buffer pH 7.0 was used in place of solvent. The organic solvents used were dimethyl sulfoxide (-1.3), isopropyl acetate (1.3), chloroform (2.0), 2-octanol (3.1), 1-decanol (4.1), decane (5.8), and n-hexadecane (8.8). All effects were determined by using olive oil as lipase substrate and colorimetric assay of activity. To detect the occurrence of any non-enzymatic hydrolysis controls without enzyme were used,

### Statistical analysis

For statistical analysis, a standard deviation for each experimental result was calculated using the Spreadsheet available in Microsoft Excel.

### 3D structure prediction

The PSI-BLAST at the National Centre for Biotechnology Information (http://www.ncbi.nlm.nih.gov/BLAST) was conducted to search for a suitable crystal structure in the protein structure database for use as a template. The 3D structure of HZ lipase was predicted by homology modeling through YASARA structure (10.4.11) using the chain 'A' of 3D structures of 2DSN (T1 lipase), 1KU0 (L1 lipase) and 1JI3 (P1 lipase) as templates.

An unrestrained high-resolution refinement with explicit solvent molecules was run, using YASARA 03 force fields. The result was validated by the YASARA program to ensure that the refinement did not move the model in the wrong direction. Based on the templates used to predict the structure, for each template, five models were built. To refine the geometry and orientation of the binding model, the highest ranked models for each template were subsequently refined using the YASARA program (http://www.yasara.org).

The predicted model was evaluated using PROCHECK (http://www.ebi.ac.uk/thornton-srv/software/PROCHECK/), Verify 3D (http://nihserver.mbi.ucla.edu/Verify_3D/) and ERRAT (http://nihserver.mbi.ucla.edu/ERRATv2/) programs.

### Ion pair/salt bridge analysis

The ion-pair analysis of HZ lipases was performed by Evaluating the Salt BRIdges in Proteins (ESBRI) software.

## Results

### Isolation and identification of HZ lipase gene

Construction of the genomic DNA library of *A*. *thermoaerophilus* strain HZ to isolate the thermostable and organic solvent-tolerant HZ lipase gene was performed through shotgun cloning. Different restriction enzymes (RE) such as *Mbo*I, *Hind*III, *Pst*I and *Eco*R1 were used to generate partially-digested DNA fragments for library construction. However, *Eco*R1 was the only RE that produced positive clones. For the genomic DNA library study, around 4000 recombinant colonies containing plasmids with inserted gene were produced. Among the white color colonies, nine positive colonies produced a halo on the tributyrin- ampicillin agar plate after 16 h. Since *E*. *coli* cells were grown for more than 16 h, cells began to lyse and the enzyme was released and produced a halo zone around the colony due to the hydrolysis of tributyrin in the plate. After sub-culturing, four colonies had grown and plasmid analysis showed that all the four colonies contained the same size of DNA insert (~ 8.0 kb).

The recombinant plasmid that contained 8.0 kb DNA insert was sequenced using pUC19 universal primers. The primer walking process was continued until generated sequence data covered the entire DNA insert. After each sequencing step, a new pair of primers was designed to amplify the remaining DNA insert. To amplify the whole 8.0 kb DNA insert, three sets of primers were designed.

**Fig 1:**
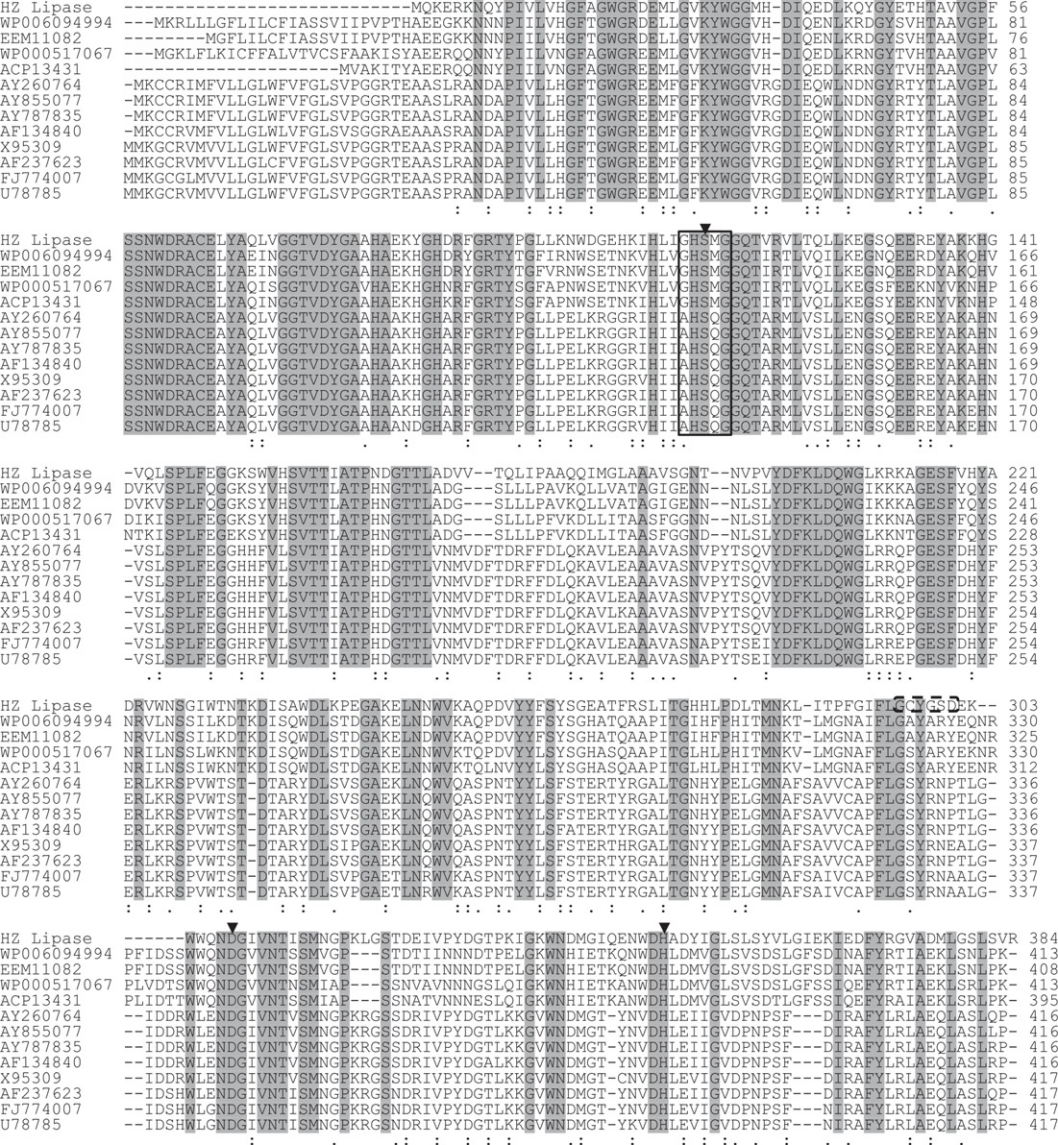
Multiple amino acid sequence alignment of HZ lipase and other thermostable lipases from subfamily I.5. The accession numbers of the aligned sequences are for the following organisms: WP_006094994, *Bacillus pseudomycoides* DSM 12442; EEM11082, *Bacillus mycoides* Rock3-17; WP_000517067, *Bacillus cereus* B4264; ACP13431, *Bacillus anthracis* CDC684; AY260764, *Geobacillus zalihae* strain T1; AY855077, *Bacillus* sp. L2; AY787835, *Bacillus* sp. 42; AF134840, *Bacillus thermoleovorans*; X95309, Bacillus thermocatenulatus; AF237623, *Bacillus stearothermophilus* P1; FJ774007, *Geobacillus* sp. NTU 03; U78785, Bacillus stearothermophilus *L1*. *Fully conserved residues are indicated by a dark gray background*. Open box is indicated conserved pentapeptide of bacterial lipases. The potential Ca^2+^ binding site in HZ lipase is boxed by a discontinuous line. *Symbols*: ▼amino acid forming a catalytic triad,: conservation of strong groups,. conservation of weak groups.

A BLAST search for the deduced nucleotide sequence revealed that the DNA fragment encoding lipase gene had 71% identity to putative lipase from *Bacillus weihenstephanensis* KBAB4 [CP000903]. The closely characterized lipases to HZ lipase were from thermostable *Bacillus* and *Geobacillus* lipases belonging to subfamily I.5. Based on the prediction of ORF finder through 3000 bp of submitted DNA fragment, HZ lipase contained an open reading frame of 1155 bp, which codes for 384 amino acids. The deduced molecular mass and pI were calculated to be 43.0 kDa and 5.74, respectively. The Shine-Dalgarno sequence, -35 and -10 promoter regions upstream from the initiation codon (ATG) at position 1 were predicted online (http.www.fruitfly.orseq_toolspromoter.html). Submitting the HZ lipase gene in the SignalP V4.1 World Wide Web server (http://www.cbs.dtu.dk/services/SignalP/) predicted that HZ lipase gene contained no signal peptide cleavage site unlike the other thermostable lipase gene in family I.5 [18]. Therefore, the ORF with 384 amino acids was deduced to be a mature lipase (S1 Fig). Multiple sequence alignments of all the 384 amino acids of HZ lipase with the closely related sequences of thermostable lipases revealed the lipase-conserved catalytic triad residues Asp (308) and His (350) and the catalytic nucleophile Ser (113) in the consensus pentapeptide GHSMG. This pentapeptide is conserved among microbial and mammalian lipases, but not in thermostable lipases of subfamily I.5 (Fig 1). Furthermore, some lipases included the Ca^2+^-binding motif, GXXGXD, in their protein sequences such as lipA of *Acinetobacter* sp. RAG-1 [19] and *Staphylococcus epidermidis* lipase [20]. The Ca^2+^-binding motif was found in HZ lipase sequence (GCYGSD), indicating that HZ lipase could be a Ca^2+^-dependent lipase (Fig 1).

### Gene analysis of HZ lipase

To analyze the HZ lipase gene, 17 lipases from various sources of psychrophilic, mesophilic and thermophilic were used to compare with the HZ lipase gene. As a preliminary determination of evolutionary relationship of the protein origin, the G+C content of microbial lipases was analyzed (Table 1). The G+C content of HZ lipase was 43.0%, and almost similar to the G+C content of *B*. *licheniformis* (42.7%) [21], which is a mesophilic lipase.

**Table 1:** Family, G+C content, Clustal distance matrix, amino acid composition and molecular weight of various lipases derived from different bacteria sources.

Thermal characteristic	Lipases	Family	G+C conten %	Clustal distance matrix	Amino acid composition				Molecular weight (kDa)
					Total amino acid	Charged residues %	Hydrophobic residues%	Uncharged residues%	
	***Aneurinibacillus thermoaerophilus* strain HZ (GU272057)**	**I.9**	**43.0**	**-**	**384**	**25**	**38.5**	**36.5**	**42.97**
	*Bacillus pseudomycoides* DSM 12442 (WP_006094994)	I.9	38.1	0.367	413	23.7	38.3	38.0	45.94
**Thermophilic**	*Geobacillus* sp. NTU 03 (FJ774007)	I.5	55.8	0.418	417	22.8	41.7	35.5	46.21
	*Bacillus stearothermophilus* P1 (AF237623)	I.5	55.0	0.418	417	22.5	41.2	36.2	46.26
	*Bacillus* sp. 42 (AY787835)	I.5	54.8	0.421	416	23.1	41.3	35.6	46.35
	*Geobacillus zalihae* strain T1 (AY260764)	I.5	54.7	0.421	416	22.8	41.3	35.8	46.33
	*Bacillus* sp. L2 (AY855077)	I.5	54.6	0.421	416	22.8	41.6	35.6	46.31
	*Bacillus thermoleovorans* (AF134840)	I.5	54.8	0.429	416	22.8	41.8	35.3	46.31
**Mesophilic**	*Staphylococcus haemolyticus* (AF096928)	I.6	33.8	0.611	711	27.4	30.4	42.2	80.18
	*Staphylococcus epidermidis* (AF090142)	I.6	36.0	0.629	643	26.7	28.8	44.5	72.20
	*Proteus vulgaris* (U33845)	I.1	37.7	0.775	290	25.2	42.1	32.8	31.67
	*Pseudomonas luteola* (AF050153)	I.2	65.9	0.757	360	14.4	45.0	40.6	37.19
	*Pseudomonas aeruginosa* (D50587)	I.1	65.9	0.820	311	16.4	42.1	41.5	32.72
	*Bacillus licheniformis* (AJ297356)	I.4	42.7	0.849	213	19.2	42.7	38.0	22.74
**Psychrophilic**	*Pseudomonas* sp. B11-1 (AF034088)	IV	62.5	0.926	308	22.1	49.7	28.2	33.71
	*Streptomyces albus* (U03114)	III	72.7	0.906	304	18.1	43.1	38.8	31.98
	*Moraxella* L1 (X53053)	III	45.7	0.913	319	21.9	42.6	35.4	34.62

The distance matrix was generated based on the multiple sequences alignment of Clustal W1.81 using CLUSTALDIST Biology Workbench. The calculated distances showed that the distance matrix of HZ lipase to other thermostable lipases is around 0.4, while it is 0.6 to 0.8 in mesophilic lipases and 0.9 in psychrophilic lipases (Table 1).

The amino acid composition of HZ lipase was analyzed with the ProtParam tool of the Expasy molecular biology (web.expasy.org/protparam/). Out of the 384 amino acid residues in the deduced sequence of the HZ lipase, the total charged residues (Arg, Lys, His, Asp and Glu) are 96 amino acids (25%). HZ lipase contains148 hydrophobic residues (Ala, Val, Ile, Leu, Met, Phe, Trp, Pro), which corresponds to 38.5% of the total amino acids encountered. The total uncharged polar residues (Ser, Thr, Asn, Gln, Cys, Gly and Tyr) are 140 amino acids (36.5%) of the total amino acid encountered. Comparison of total charged, hydrophobic and uncharged polar amino acid residues for various lipases is shown in Table 1.

The amino acid compositions of lipases vary among the families, and result in different molecular weights (MW) from 22.74 kDa (*B*. *licheniformis*, AJ297356) to 80.18 kDa (*S*. *haemolyticus*, AF096928) [21,22]. HZ lipase with 42.97 kDa has lower molecular weight than other thermostable lipases.

**Fig 2:**
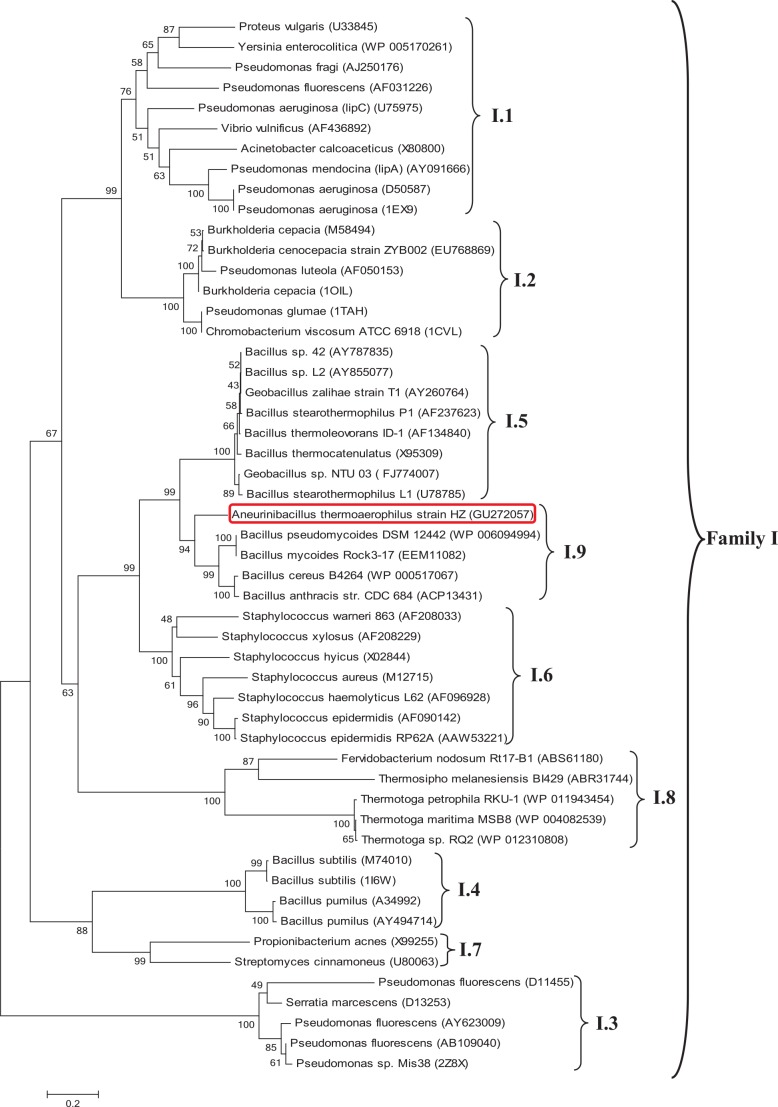
Phylogenetic relationships of lipases homologues to HZ lipase. The sequences were aligned with ClustalW; distance matrices and phylogenetic trees were constructed based on Neighbor-Joining algorithm in the MEGA 6.0 computer software program, using Poisson correction model. The numbers at the nodes are bootstrap confidence values expressed at percentages of 1000 bootstrap replications. The bar scale, 0.2, indicates branch length proportional to estimated divergence along each branch.

The phylogenetic tree of HZ lipase revealed the evolutionary relationship of the HZ lipase gene with other members of lipases from family I. Fig 2 shows a tree of lipases from different sub-families of family I generated by MEGA 6.0 software.

Phylogenetic analysis showed that HZ lipase is closely related to uncharacterized lipases from *B*. *pseudomycoides* DSM 12442 (WP_006094994), *B*. *mycoides* Rock3-17 (EEM11082), *B*. *cereus* B4264 (WP_000517067) and *B*. *anthracis* str. CDC684 (ACP13431) that were previously identified in bacterial genome sequences with around 62–60% identity. HZ lipase is moderately related to sub-families I.5 and I.6 of family I with around 55 and 40% identity, respectively. The HZ lipase is distantly related to lipases from sub-families I.1, I.2 and I.8 that stand in the same main branch with them.

### Effect of temperature on activity and stability of HZ lipase

**Fig 3:**
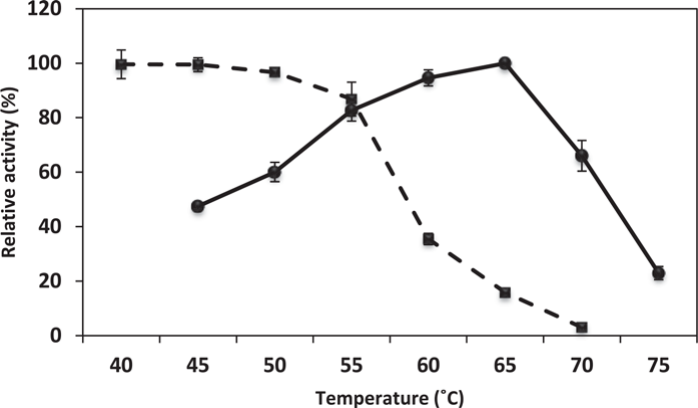
Effect of temperature on HZ lipase activity and stability. ● Temperature-activity curve: results are expressed as a percentage of the maximal activity.■ Temperature-stability curve: results are expressed as a percentage of the residual activity. SD is indicated as error bars. When the error bar cannot be seen, the deviation is less than the size of the symbol.

The effect of temperature on HZ lipase activity was tested from 45 to 75°C. As presented in Fig 3, the enzyme showed its maximal activity at 65°C with olive oil as substrate and kept more than 60% of this activity on a broad T range (between 50 and 70°C). The enzyme was shown to be highly stable for at least 1 h at T≤ 50°C (Fig 3). Beyond this temperature there was inactivation of no more than 13% after 1 h incubation at 55°C.

### Effect of organic solvents on HZ lipase

**Fig 4:**
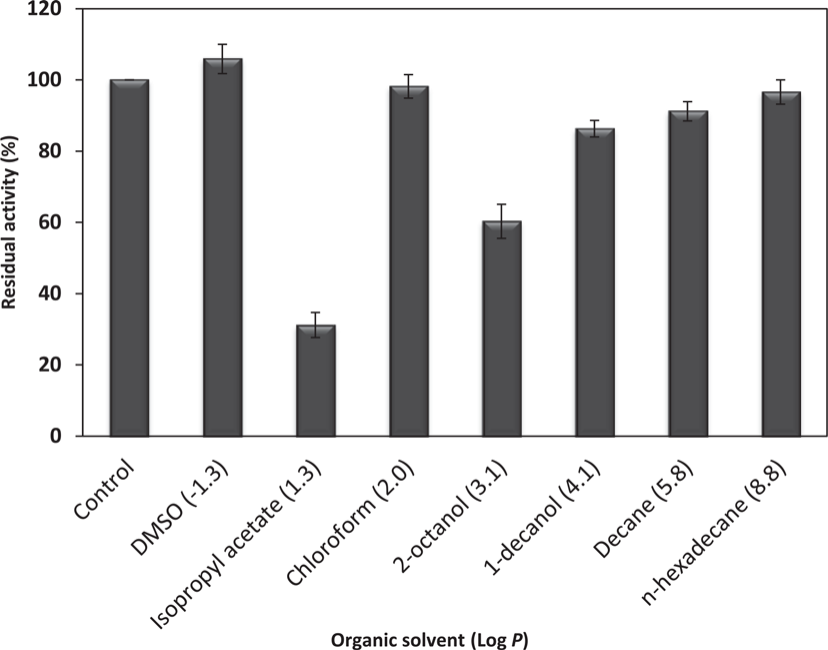
Effect of organic solvents on stability of the recombinant HZ lipase. 25% (v/v) of organic solvents were added to the intracellular enzyme and incubated at 50°C with 200 rpm shaking for 30 min. SD is indicated as error bars. When the error bar cannot be seen, the deviation is less than the size of the symbol.

Fig 4 reveals that the recombinant HZ lipase was stable in the organic solvents with log *P* values of -1.3 to 8.8 and the enzyme activity was in excess of 90% of that in the absence of an organic solvent, except ester and/or alcohol organic solvents such as isopropyl acetate, 2-octanol and 1-decanol. The higher lipase activity was shown by HZ lipase in the presence of dimethyl sulfoxide (Log *P* -1.3).

### Prediction of three-dimensional structure

The result of PSI-Blast showed that the HZ lipase exhibited high similarity with the crystal structures of thermostable lipases from subfamily I.5 with sequence identities of 57% (S1 Table). As the model was built by the YASARA program, the crystal structures of thermostable lipase, not the mutants, were chosen as the templates to extrapolate structure information of the HZ lipase. The selection was based on high sequence identity encountered from the same family of enzyme as they shared high similarity in their functions (see supplementary information) [23].

YASARA Structure involves a complete homology modeling module that fully and automatically takes all the steps from an amino acid sequence to a refined reliable model using a CASP (Critical Assessment of protein Structure Prediction) approved protocol [24].

**Fig 5:**
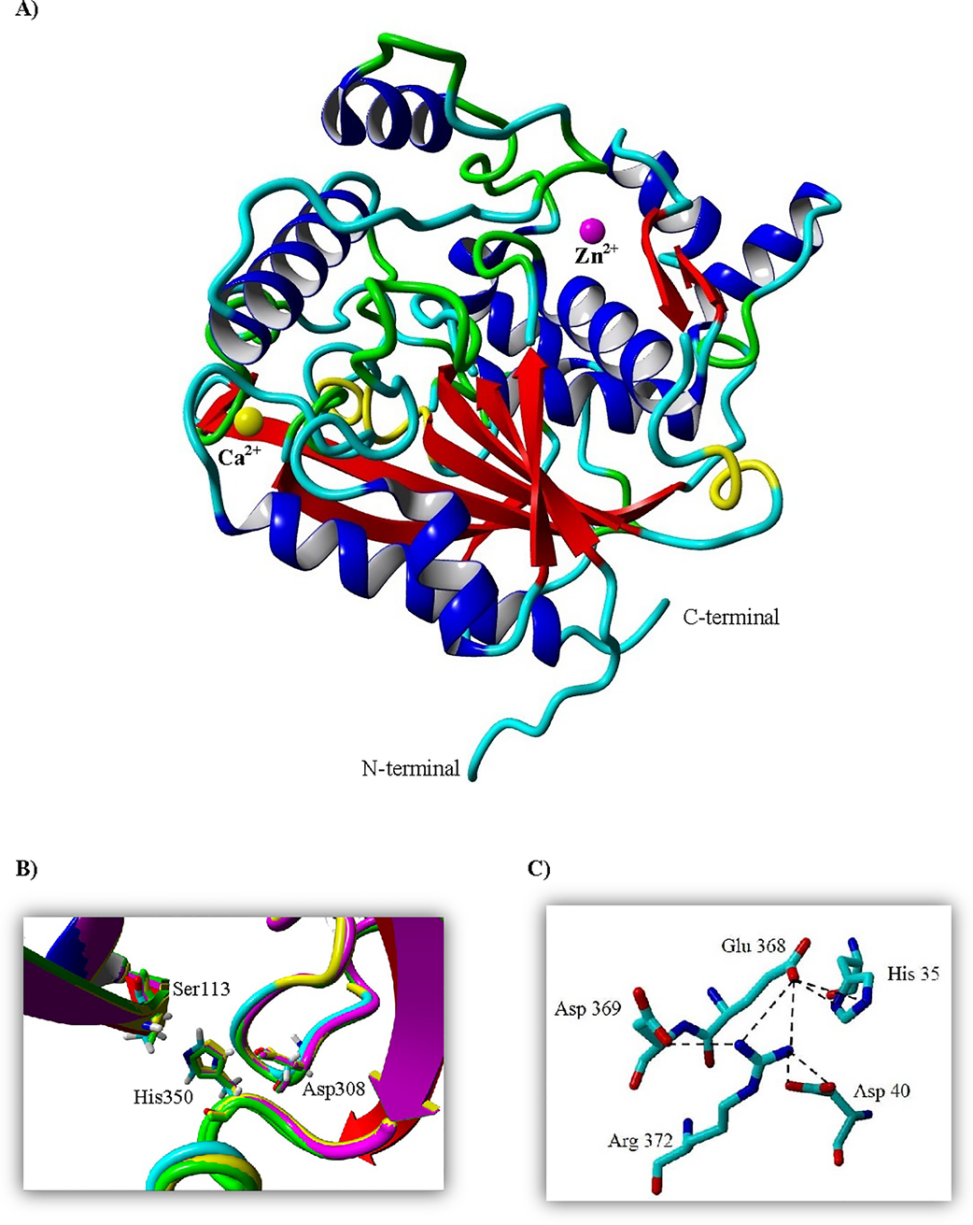
Three-dimensional structure of HZ lipase by using homology modeling. **A) Predicted 3D structure of HZ lipase using 3D structure of 2DSN, 1KU0 and 1JI3 by YASARA in linear ribbon.** Metal ions, Ca^2+^and Zn^2+^, are shown as solid circles. 10 α-helices (blue), 13 β-sheets (red), turn (yellow) and 310-helix (green) are arranged in a single domain. **B) The predicted catalytic triad of the HZ lipase superposed with T1 (magenta), L1 (yellow) and P1 (green) lipases.** It comprised Ser 113, Asp 308 and His 350, similar to other lipases. **C) Ion-pair networks in predicted HZ lipase structure.**

The predicted model of HZ lipase contained one main region consisting of 10 α-helices and 13 β-sheets in the folded protein (Fig 5A). The β-sheets were in the core region surrounded by α-helices to form a typical α/β hydrolase. Three catalytic residues include Ser113, His308 and Asp350. YASARA predicted two metal ions, Zn^2+^ and Ca^2+^, in HZ lipase structure which is common in all crystal structures of thermostable lipases in subfamily I.5 (Fig 5B). The validity of the structure was confirmed by three different softwares. The Ramachandran plot of the predicted HZ lipase structure showed that 92.6% (300) of the residue lie in the most-favored region, with 6.8% (22) of residues in the additional allowed region and 0.6% (2) of residues in generously allowed regions (S2 Fig). The compatibility of segments of the HZ lipase amino acid sequence with their 3D structure in HZ lipase was assessed by plotting the 3D-1D averaged score against sequence number (data not shown). In the predicted structure, the 3D-1D averaged score fluctuated from -0.04 to 0.71, 96.9% of the total residues of the predicted model have 3D-1D averaged score of more than 0.1, and average score of 3aa were lower than zero. Based on the analysis by ERRAT, the predicted model was significantly acceptable according to 98.4% overall quality factor (data not shown), because the structure of the model was derived from 3 crystal structure templates with good resolution.

### Ion pair/salt bridge analysis

The predicted model was analyzed for possible ion pairs with distances of less than 4 Å between oppositely charged residues. The results showed that some different amino acids are involved in ion-pair formation in the HZ lipase and the templates. In HZ lipase, there are 28 charged residues involved in 24 ion-pair interactions. Furthermore, HZ lipase contained a large ion-pair network made up of five different amino acid residues. His 35, Asp40, Glu368, Asp369 and Arg 372 are connected by seven ion-pairs (Fig 5C).

## Discussion

The evolution of gene families can be understood by the analysis of sequence similarities in distantly related proteins. Arpigny and Jaeger [7] had classified bacterial lipolytic enzymes based on their amino acid sequences and biological properties. Thermostable lipases from thermophilic bacteria are grouped together into subfamily 5 of family I, which consists of true lipases. Thermostable lipases are highly conserved within the sequences of their ORF. Therefore, even with the limited information on a thermostable lipase gene from closely related thermophilic *Bacillus* and *Geobacillus* spp., it should be possible to isolate the lipase gene derived from a closely related member of the family. However, in preliminary attempts to isolate the HZ lipase gene, the degenerative PCR primers, which were designed based on the conserved nucleotide sequences of the thermostable and mesophilic lipase genes from *Bacillus* and *Geobacillus* spp, could not amplify a lipase gene. Hence, genomic DNA library construction was carried out and successfully isolated the lipase gene from *A*. *thermoaerophilus* strain HZ.

The BLAST search of the deduced amino acid sequence revealed the relatively high identities between HZ lipase gene and other *Bacillus* lipases, including a lipase from *B*. *pseudomycoides* DSM 12442 [WP_006094994] (62%), a lipase from *B*. *mycoides* Rock3-17 [EEM11082] (62%) and a lipase from *B*. *anthracis* strain Ames [WP_000257468] (60%), which were previously identified only in bacterial genome sequences. However, none of them has been characterized to date. A moderate identity was observed for lipases from thermophilic bacteria, such as the thermostable lipase. Multiple sequence alignments of HZ lipase with other thermostable lipases from family I.5 demonstrated the differences in the amino acid composition and the consensus pentapeptide of HZ lipase (Fig 1), which could be one of the reasons supporting the classification of HZ lipase as a new member of the true lipases. The amino acid sequence of the HZ lipase from *A*. *thermoaerophilus* strain HZ is found in the GenBank database under the protein ID ADC84241.

Detailed analysis of the HZ lipase gene revealed significant differences of this gene in comparison with other microbial thermostable lipases present in family I. To broaden the comparison, lipases from various sources of psychrophilic, mesophilic and thermophilic origin from different families were compared. The G+C content of a gene encoding a protein may determine the origin of the protein. However, it is not always the case as two very different organisms can have similar or even identical GC ratios [25]. The closest lipase with 62% maximum identity to the HZ lipase was from *B*. *pseudomycoides* DSM 12442 with the G+C content of 38.1%. The thermostable lipases from *Geobacillus* spp. and *Bacillus* spp. showed fairly high G+C ratios (54.6 to 55.8%) compared to the HZ lipase [11,26–30]. The lipases from *Pseudomonas* spp. [31–33] and *S*. *albus* [34] possessed a higher ratios of G+C compared to other lipase genes (62.5 to 72.7%). Furthermore, the divergence among the lipases can be seen as the distance matrix describing the evolutionary relationship of lipases. The calculated distances showed that the HZ lipase was more related to the thermostable lipases compared to the mesophilic or psychrophilic lipases.

Moreover, analysis of amino acid sequence showed that the number of charged amino acid residues of the thermostable HZ lipase (25%) is almost higher than other thermostable lipases (22.5 to 23.1%), even though, the ORF of HZ lipase does not have a signal peptide. Also, the number of charged amino acid residues of HZ lipase is higher than the lipases from mesophilic and psychrophilic sources, except for the lipases from *S*. *haemolyticus* [AF096928] [22], *S*. *epidermidis* [AF090142] [35] and *P*. *vulgaris* [U33845] [36], which contain 27.4, 26.7 and 25.2% charged residues, respectively. Most lipases have high proportions of hydrophobic residues of above 40%, but HZ lipase and lipase from *B*. *pseudomycoides* DSM 12442 [WP_006094994] have lower fractions of hydrophobic residues, 38.54 and 38.26%, respectively. A few studies have reported that some biological functions of protein are associated with the relative identity by Blast search, multiple sequence alignment, sequence motif, G+C content, distance matrix, and amino acid composition [25,37]. Therefore, HZ lipase could be a new thermostable protein gene which has different sequence motif, G+C content and amino acid composition compared to other thermostable lipases from *Geobacillus* spp. and *Bacillus* spp..

In analyzing sequences of proteins, construction of phylogenetic trees conveys information regarding the evolutionary relationship among a group of genes or species. In particular, phylogenetic trees can be applied to coordinate the information about protein sequence similarities with the evolution of corresponding species. Jaeger and colleagues previously reported the broad classification of bacterial lipolytic enzymes, mainly based on a comparison of their amino acid sequences and their biological properties [7,8], which allowed the classification of HZ lipase. For the phylogenetic analysis, 47 bacterial lipolytic enzymes representing eight different subfamilies of family I were selected. As shown in Fig 2, HZ lipase and the four putative bacterial lipases did not belong to any of the known lipases in family I. Therefore, we suggest that they comprise a new subfamily I.9 of bacterial lipolytic enzymes.

The important feature of thermostable lipases from *Bacillus* spp. and *Geobacillus* spp. belonging to family I.5 is that they share the same conserved pentapeptide AXSXG. Probably lipases from family I.5 have a common ancestor. Nevertheless, the conserved pentapeptide in HZ lipase contains Gly instead of Ala in the first residue, as do certain mesophilic lipases, but HZ lipase has relatively high optimum temperature.

The optimal temperature for recombinant HZ lipase was at 65°C. Despite the strong dependence of lipase activity on assay temperature, a temperature increase to 65°C failed to follow this trend due probably to the flexibility of increased enzyme or to a change in the conformation of the protein, resulting in loose bounding to the substrate [38]. In addition, the recombinant HZ lipase was still stable at high temperature up to 55°C for 1 h incubation. BTL2 lipase from *B*. *thermocatenulatus* BTL2 (X95309), which had 56% identity to HZ lipase, showed maximum temperature activity at 65°C. However, the enzyme was stable up to 50°C when incubated for 30 minutes and above this temperature, the residual activity decreased steeply to 61 and 18% at 60 and 70°C [39].

Ironically, there are many reports on the characterization of microbial lipases but very few lipases have been reported having both characteristics; thermostable and organic solvent-tolerant suitable for harsh industrial processes. Most organic solvent-tolerant lipases are stable in either very polar or non-polar organic solvent, but not in both [40]. *Bacillus* lipases are mostly stable in non-polar organic solvents. The polar water-miscible solvents could destabilize the enzyme by removing the solvation water from the enzyme [41]. However, there could be a relationship between polar solvents stability with substrate inhibition in the synthesis of flavor esters and the production of biodiesel [38]. The recombinant HZ lipase showed high stability in the presence of very polar organic solvent (DMSO) and non-polar solvent (n-hexadecane) which makes it an ideal protein for fundamental study and industrial use.

The three-dimensional structure of a protein describes the folding of its secondary structural elements and specifies the positions of each atom in the protein, including those of its side chains. The homology models can hypothesize structure-function relationships [42]. The prediction of HZ lipase structure was done based on the respective amino acid sequences. Homology models built on the basis of a significant sequence identity between target and templates, above 50–60% are certainly accurate in their overall structure and can be reliably used to analyze the conserved regions of the protein, such as its active site [23].

The catalytic residues were placed at highly conserved geometry in the loops on one side of the sheet and an oxyanion hole formed by two backbone amides of residues in the N-terminal region of the HZ lipase and the C-terminal neighbor of the catalytic serine (Fig 5). The Ser113 appeared to be in the pentapeptide GXSXG motifs situated at the sharp turn between the β-strand and α-helix that resembles the nucleophilic elbow, which usually exists in the structural family of α/β hydrolases. The function of the catalytic serine is suggested to be aided by the oxyanion hole that stabilizes the negative charge generated during the nucleophilic attack by the Ser Oγ [43].

There are several parameters related to enzyme thermostability, one of which is stabilization by ligands. The two metals, Zn^2+^ and Ca^2+^, are identified in the predicted structure. Metal ions assisting in stabilizing the protein structure, may cause a conformational change when bound, and/or participate in catalysis [44]. Metal ions play significant roles in influencing the structure and function of lipases [45,46] and they are especially important in thermostable enzymes. These ions are bound to specific binding sites on the surface of molecules and play a structural role, keeping together by coordination and fix the conformation of certain flexible segments of the polypeptide chain [38].

P1 and L1 lipases were the first lipase structures found to possess these metal ions which later have been found to be conserved among other thermostable lipases. Studies have indicated that by eliminating these metal ions from the structure, the thermostability of the enzymes will decrease [47–50].

The predicted HZ lipase structure was superposed to the crystal structure of T1 lipase (2DSN) with RMSD of 4.05 Å over 112 matched alpha carbon atoms. Considering the 57% similarity in sequence alignment between HZ lipase and T1 lipase, and 167 amino acids difference between the two lipases, in homology modeling, the current value of RMSD is acceptable [51]. Essential for interpreting 3D protein models is the estimation of their accuracy, both the overall accuracy and the accuracy in the individual regions of a model. The quality of the predicted model structure is strongly dependent on the accuracy of the template structure used (see the supporting information) [52]. As shown in Table 2, the quality of the predicted model of HZ lipase and the templates has been evaluated using three different types of criteria. Based on the evaluation results, the quality of the backbone and the non-bonded interaction between atoms were at satisfactory levels. The folding of predicted HZ lipase compared to the templates could be acceptable due to the only 57% sequence similarity between the HZ lipase and templates.

**Table 2:** Summary of evaluation of predicted model of HZ lipase and T1, L1 and P1 lipases structure.

Model	PROCHEK(Ramachandran plot)		Verify 3D		ERRAT
	Residue in most favored regions	Residue in disallowed region	3D-1D average score	Residue below zero	Overall quality factor
Predicted HZ lipase	300aa-92.6%	-	-0.04 to 0.71	3aa	98.4%
T1 lipase (2DSN)	298aa-90.3%	1aa-0.3%	0.29 to 0.77	-	97.6%
L1 lipase (1KU0)	294aa-89.4%	-	0.17 to 0.77	-	93.9%
P1 lipase (1JI3)	300aa-90.6%	1aa-0.3%	0.2 to 0.79	-	96.6%

Meanwhile, the HZ lipase model was analyzed for possible ion-pair interactions and network. A substantial rise in the number of ion-pairs has been indicated for the majority of structures of thermostable proteins [53]. Also, the ion-pair interactions have enhanced the forces that bind the monomers in the protein structure. The HZ lipase contained 24 ion-pair interactions. In comparison, in T1, L1 and P1 lipases, 34, 26 and 36, charged residues are involved in 29, 21 and 33 ion-pair interactions, respectively. Even though, the percentage of charged residue in HZ lipase was higher than T1 and L1 lipases, the number of ion-pair interactions was lower.

For example, the crystal structure of T1 lipase (only the mature part of protein) contains 23.45% charged amino acid residue, while 25% charged residues are present in the total amino acid composition of HZ lipase. On the other hand, Chakravorty et al.[54] reported that stability of protein in the presence of organic solvents was related to the surface charge residue of the protein. The surface charge residue could establish the repulsive forces to exclude solvent molecules from interacting with the surface and prohibit the same from gaining entry to the protein core, thus stabilizing the active conformation of the protein. Therefore, free charged residue in the surface of HZ lipase might be the reason for stabilizing of protein in the presence of organic solvents over a wide range of log *P*. S3 Fig shows the free charged amino acid residues on predicted 3D structure of HZ lipase which mostly are on the surface of HZ lipase.

Furthermore, ion-pair networks formed on the surface of the protein subunits buried at the interdomain and intersubunit interfaces may be a significant stabilizing feature related to the adaptation of enzymes to extreme temperatures [55]. Table 3 shows the amino acid residues that are involved in ion-pair networks in the HZ lipase and the three templates. In the ion-pair network of the templates (T1, L1, P1 lipases), Arg92, Asp205 and Asp209 are involved but not in HZ lipase ion-pair network, whereas these three amino acids are in the conserved regions of thermostable lipases of subfamily I.5 (Fig 1). The above results indicate that the side chains of charged amino acid residues in the predicted model are positionally different from those of the templates.

**Table 3:** The number of charged residues involved in ion-pair network formation in Predicted model of HZ and its templates structure.

3D Model	Residue 1	Position	Residue 2	Position	Distance
**Predicted HZ lipase**	His 35	ND1	Glu 368	OE1	3.85
		NE2		OE1	3.66
	Arg 372	NH1	Asp 40	OD1	2.97
		NH1		OD2	3.25
		NH1	Glu 368	OE1	3.15
		NH2		OE1	3.92
		NH2	Asp 369	OD1	2.96
**T1 lipase (2DSN)**	Arg 92	NH1	Asp 205	OD2	3.24
		NH2		OD1	3.60
		NH2		OD2	3.07
		NH2	Asp 209	OD1	2.87
		NH2		OD2	3.80
	Lys 229	NZ	Asp 178	OD1	2.70
		NZ	Glu 226	OE1	3.86
	Arg 230	NH1	Glu 226	OE1	3.07
		NH2		OE1	3.79
**L1 lipase (1KU0)**	Arg 21	NH1	Asp 36	OD1	3.18
		NH1		OD2	3.02
		NH1	Glu 38	OE1	3.38
		NH2	Asp 36	OD1	2.72
		NH2		OD2	3.97
		NH2	Glu 38	OE1	3.77
	Lys 28	NZ	Asp 36	OD1	3.46
	Arg 92	NH1	Asp 205	OD1	2.90
		NH2		OD1	2.70
		NH2		OD2	3.34
		NH2	Asp 209	OD1	2.93
		NH2		OD2	3.97
	Arg 214	NH2	Asp 209	OD2	3.59
**P1 lipase (1JI3)**	Arg 92	NH1	Asp 205	OD2	2.73
		NH2		OD1	3.07
		NH2		OD2	2.47
		NH2	Asp 209	OD1	3.52
		NH2		OD2	3.89

**Note:** Atoms are identified using PDB file format nomenclature, as follows: NE, OE = Nitrogen/Oxygen, Epsilon; NZ = Nitrogen, Zeta; NH = Nitrogen, Eta.

In conclusion, thermostable and organic solvent-tolerant HZ lipase gene is an important enzyme, which can withstand elevated temperatures and tolerate harsh organic solvents. The gene analysis showed that HZ lipase belong to the family I, but new subfamily, 1.9 as the sequence homology, clustal distance matrix and amino acid composition showed differences from the other thermostable lipases represented in family I. In addition, based on the constructed model, the possibility of ion-pairs in the protein structure was analyzed and the relation between the charged residues was highlighted. Moreover, the differences in the sequence and structure of this new lipase make it valuable in fundamental study.

## Supporting Information

S1 Fig The nucleotide and amino acid sequences of the HZ lipase gene from *A*. *thermoaerophilus* strain HZ. The predicted promoter region (-10 and -35 promoter) and ribosome binding site (RBS) are underlined. The transcription start is shown in larger font. The inverted repeat sequence downstream of the HZ lipase gene is indicated using horizontal arrows. A pentapeptide conserved among thermostable lipases is indicated by the box. The asterisks indicate the primers used for full length HZ lipase gene cloning. The HZ lipase sequence has been submitted to the GenBank database under the accession number GU272057.
(DOCX)

S2 Fig Ramachandran plot of predicted HZ lipase structure. The most favored region (red), additional allowed region (orange-brown), generously allowed region (dark yellow) and disallowed region (light yellow) were used to evaluate the quality of the structure.
(DOCX)

S3 Fig Predicted 3D structure of HZ lipase showing abundance of free charged amino acid residues on the model. Only the negative and positive charged side chains are shown, colored red and blue, respectively. The core structure backbone is shown in gray.
(DOCX)

S1 Table PSI blast of HZ lipase gene from *A*. *thermoaerophilus* strain HZ. (DOCX)

S1 Text Template search and selection for HZ lipase structure prediction. (DOCX)

S2 Text Evaluation of the predicted HZ lipase structure. (DOCX)
