# Supplementary material for: Analysis of Comparative Sequence and Genomic Data to Verify Phylogenetic Relationship and Explore a New Subfamily of Bacterial Lipases
Source: PLoS One. 2016 Mar 2;11(3):e0149851. doi: 10.1371/journal.pone.0149851 (PMC4774917; doi:10.1371/journal.pone.0149851)
Supplement: S1 Fig — The predicted promoter region (-10 and -35 promoter) and ribosome binding site (RBS) are underlined. The transcription start is shown in larger font. The inverted repeat sequence downstream of the HZ lipase gene is indicated using horizontal arrows. A pentapeptide conserved among thermostable lipases is indicated by the box. The asterisks indicate the primers used for full length HZ lipase gene cloning. The HZ lipase sequence has been submitted to the GenBank database under the accession number GU272057. (DOCX) [file pone.0149851.s001.docx]

**S1 Fig.** **The nucleotide and amino acid sequences of the HZ lipase gene from *A. thermoaerophilus* strain HZ.** The predicted promoter region (-10 and -35 promoter) and ribosome binding site (RBS) are underlined. The transcription start is shown in larger font. The inverted repeat sequence downstream of the HZ lipase gene is indicated using horizontal arrows. A pentapeptide conserved among thermostable lipases is indicated by the box. The asterisks indicate the primers used for full length HZ lipase gene cloning. The HZ lipase sequence has been submitted to the GenBank database under the accession number GU272057.

AACAGGTAAGGTTTATGTAACAACTGAATTGATTCAGAAATTACGCCGAATTTTCGGCGT

TTT**TTTATG**CCCTTTTATCTTGGGCAAC**TCAAAT**AAACG**T**AAAAA**AGGAGC**TGTTTTATC

******************

-35 -10 RBS

ATGCAAAAGGAAAGAAAAAATCAATATCCAATCGTTCTAGTTCATGGGTTTGCAGGTTGG

**********************

M Q K E R K N Q Y P I V L V H G F A G W

GGAAGAGATGAAATGCTGGGTGTAAAGTACTGGGGTGGAATGCATGACATTCAAGAGGAT

G R D E M L G V K Y W G G M H D I Q E D

TTGAAACAGTATGGTTACGAAACACACACTGCGGTAGTAGGACCGTTTTCAAGTAACTGG

L K Q Y G Y E T H T A V V G P F S S N W

GATCGTGCATGCGAATTATATGCTCAACTTGTTGGTGGAACAGTGGATTATGGTGCTGCA

D R A C E L Y A Q L V G G T V D Y G A A

CACGCTGAAAAATATGGACATGACCGGTTTGGTCGAACCTATCCTGGGCTTTTGAAGAAT

H A E K Y G H D R F G R T Y P G L L K N

TGGGATGGAGAACACAAAATCCATTTAATCGGACATAGCATGGGTGGACAGACGGTTCGT

W D G E H K I H L I G H S M G G Q T V R

GTGTTAACGCAATTGTTAAAAGAGGGAAGCCAGGAAGAACGAGAGTATGCGAAAAAGCAT

V L T Q L L K E G S Q E E R E Y A K K H

GGGGTGCAATTGTCTCCGCTATTTGAAGGTGGGAAGTCGTGGGTTCACAGTGTAACAACG

G V Q L S P L F E G G K S W V H S V T T

ATTGCAACGCCGAACGATGGTACAACGTTGGCTGATGTAGTGACACAACTCATTCCAGCA

I A T P N D G T T L A D V V T Q L I P A

GCACAACAAATTATGGGACTGGCTGCTGCGGTGTCAGGCAATACAAATGTACCGGTTTAT

A Q Q I M G L A A A V S G N T N V P V Y

GACTTCAAACTCGATCAGTGGGGATTGAAAAGAAAAGCAGGTGAATCCTTTGTGCACTAT

D F K L D Q W G L K R K A G E S F V H Y

GCGGATCGCGTATGGAATAGTGGAATTTGGACAAATACAAAAGATATTAGTGCATGGGAC

A D R V W N S G I W T N T K D I S A W D

CTCAAACCAGAAGGAGCCAAAGAGCTGAATAACTGGGTAAAAGCGCAGCCGGATGTCTAT

L K P E G A K E L N N W V K A Q P D V Y

TATTTCTCATACAGTGGAGAAGCTACATTCAGAAGTCTGATCACGGGACATCATCTCCCA

Y F S Y S G E A T F R S L I T G H H L P

GATCTAACAATGAATAAATTAATTACTCCATTTGGTATTTTCTTAGGCTGCTACGGCAGT

D L T M N K L I T P F G I F L G C Y G S

GATGAAAAGTGGTGGCAGAATGATGGGATTGTAAACACAATTTCGATGAATGGGCCGAAG

D E K W W Q N D G I V N T I S M N G P K

CTTGGCTCAACAGATGAGATTGTCCCATATGATGGAACACCTAAAATCGGAAAATGGAAT

L G S T D E I V P Y D G T P K I G K W N

GACATGGGGATTCAGGAAAACTGGGATCATGCTGATTATATTGGGCTCAGTCTTTCATAT

D M G I Q E N W D H A D Y I G L S L S Y

GTTTTGGGAATAGAAAAAATTGAAGATTTCTACCGTGGCGTTGCAGATATGCTTGGTTCA

********

V L G I E K I E D F Y R G V A D M L G S

TTATCTGTGAGATAAGAATAAAGCATGGGGTGTCTTGTAATTAACAGGGGAGGGGCCTCC

********************************

L S V R -

CCTTTTTTATGAAAAAAAGAAGATGCTACAAAGAGCATCGAGTGATGAGGGAGCTTTGAA

GACTCGCTGGTGTCAGCAGCGGGTAAAGAAGGGTAAGATATTATCAACTATAATTTTACT
