# Supplementary material for: Analysis of Comparative Sequence and Genomic Data to Verify Phylogenetic Relationship and Explore a New Subfamily of Bacterial Lipases
Source: PLoS One. 2016 Mar 2;11(3):e0149851. doi: 10.1371/journal.pone.0149851 (PMC4774917; doi:10.1371/journal.pone.0149851)

**S2 Fig. Ramachandran plot of predicted HZ lipase structure.** The most favored region (red), additional allowed region (orange-brown), generously allowed region (dark yellow) and disallowed region (light yellow) were used to evaluate the quality of the structure.


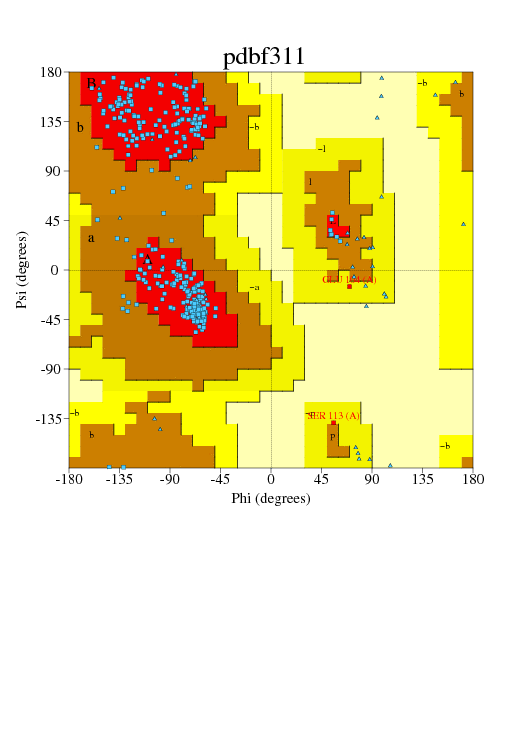

Supplement: S2 Fig — The most favored region (red), additional allowed region (orange-brown), generously allowed region (dark yellow) and disallowed region (light yellow) were used to evaluate the quality of the structure. (DOCX) [file pone.0149851.s002.docx]
