# Supplementary material for: Analysis of Comparative Sequence and Genomic Data to Verify Phylogenetic Relationship and Explore a New Subfamily of Bacterial Lipases
Source: PLoS One. 2016 Mar 2;11(3):e0149851. doi: 10.1371/journal.pone.0149851 (PMC4774917; doi:10.1371/journal.pone.0149851)

S3 Fig. Predicted 3D structure of HZ lipase showing abundance of free charged amino acid residues on the model. Only the negative and positive charged side chains are shown, colored red and blue, respectively. The core structure backbone is shown in gray.


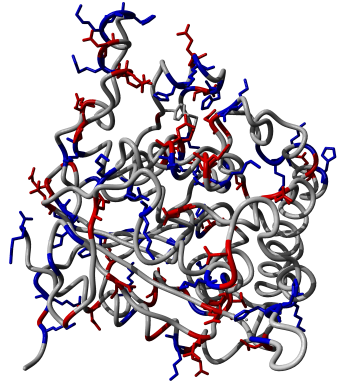

Supplement: S3 Fig — Only the negative and positive charged side chains are shown, colored red and blue, respectively. The core structure backbone is shown in gray. (DOCX) [file pone.0149851.s003.docx]
