# Supplementary material for: Analysis of Comparative Sequence and Genomic Data to Verify Phylogenetic Relationship and Explore a New Subfamily of Bacterial Lipases
Source: PLoS One. 2016 Mar 2;11(3):e0149851. doi: 10.1371/journal.pone.0149851 (PMC4774917; doi:10.1371/journal.pone.0149851)
Supplement: S1 Table — (DOCX) [file pone.0149851.s004.docx]

**S1 Table: PSI blast of HZ lipase gene from *A. thermoaerophilus* strain HZ**.

| **Crystal structure** | **Subfamily** | **Length** | **E_value** | **Identity**  **(%)** | **Resolution**  **(Å)** |
| --- | --- | --- | --- | --- | --- |
| >gi\|377656427\|pdb\|3UMJ\|A | I.5 | 387 | 8e-135 | 57 | 2.10 |
| >gi\|152148977\|pdb\|2DSN\|A | I.5 | 387 | 3e-134 | 57 | 1.50 |
| >gi\|159795726\|pdb\|2Z5G\|A | I.5 | 387 | 2e-133 | 57 | 1.80 |
| >gi\|23200288\|pdb\|1KU0\|A | I.5 | 388 | 2e-132 | 57 | 2.00 |
| >gi\|24987388\|pdb\|1JI3\|A | I.5 | 388 | 4e-132 | 57 | 2.20 |
| >gi\|374977502\|pdb\|3AUK\|A | I.5 | 389 | 1e-131 | 56 | 1.66 |
| >gi\|218681560\|pdb\|2W22\|A | I.5 | 389 | 9e-131 | 56 | 2.20 |
| >gi\|149241972\|pdb\|2HIH\|A | I.6 | 431 | 4e-82 | 40 | 2.86 |
| >gi\|2194040\|pdb\|1OIL\|A | I.2 | 320 | 4e-07 | 28 | 2.10 |

**Note:** *Geobacillus zalihae* T1 lipase D311E mutant (3UMJ), [*Geobacillus zalihae*](http://www.ncbi.nlm.nih.gov/Taxonomy/Browser/wwwtax.cgi?lvl=0&id=213419) T1 lipase (2DSN), [*Geobacillus zalihae*](http://www.ncbi.nlm.nih.gov/Taxonomy/Browser/wwwtax.cgi?lvl=0&id=213419) T1 lipase F16L mutant (2Z5G), [*Bacillus stearothermophilus* L1 lipase](http://www.pdb.org/pdb/explore/explore.do?structureId=1KU0) (1KU0), *Bacillus stearothermophilus* P1 lipase (1JI3), *Geobacillus* sp. SBS-4S lipase (3AUK), *Bacillus thermocatenulatus* BTL2 lipase (2W22), *Staphylococcus hyicus* lipase (2HIH) and *Burkholderia cepacia* lipase (1OIL).
