# Supplementary material for: Analysis of Comparative Sequence and Genomic Data to Verify Phylogenetic Relationship and Explore a New Subfamily of Bacterial Lipases
Source: PLoS One. 2016 Mar 2;11(3):e0149851. doi: 10.1371/journal.pone.0149851 (PMC4774917; doi:10.1371/journal.pone.0149851)
Supplement: S1 Text — (DOCX) [file pone.0149851.s005.docx]

#### S1 Text. Template search and selection for HZ lipase structure prediction.

Structure prediction by homology modeling is based on accurate sequence alignments between a query protein and a template protein with solved structures; hence, the prediction accuracy heavily depends on the sequence similarity between two proteins [[1](#_ENREF_1)]. The prediction of HZ lipase structure has been done on the basis of the respective amino acid sequences. HZ lipase sequence was found to be 57% identical to T1 lipase, for which the 3D structure is available. Homology models built on the basis of a significant sequence identity between target and template, above 50-60% are certainly accurate in their overall structure and can be reliably used to analyze the conserved regions of the protein, such as its active site [[1](#_ENREF_1)].

So, the critical first step in homology modeling is the identification of the best template structure. Traditionally, the selection of template structures is done by programs that detect sequence similarity only, including FASTA, BLAST, and programs based on dynamic programming methods [[2](#_ENREF_2)]. However, remotely related sequence-structure pairs need to be detected through a more sophisticated method that relies on structural information or multiple sequences from the family of interest. More sensitive methods based on multiple sequence alignment, of which PSI-BLAST constructs and performs a NCBI BLAST search with a custom, position-specific, scoring matrix, which could help to find distant evolutionary relationships. The amino acid sequence of HZ lipase with protein ID of ADC84241 was aligned using PSI-BLAST at National Center for Biotechnology Information (<http://www.ncbi.nlm.nih.gov/BLAST>). Blast was developed to perform rapid searches for homologous sequence in Protein Data Bank proteins (PDB). The result of PSI-Blast showed that the HZ lipase exhibited high similarity to the crystal structures of D311E lipase (3UMJ), T1 lipase (2DSN), T1 lipase F16L mutant (2Z5G), L1 lipase (1KU0) and P1 lipase (1JI3) with sequence identities of 57% (S1 Table).

In addition, HZ lipase shared sequence identities of 56% with *Geobacillus* sp. SBS-4S lipase (3AUK) and *Bacillus thermocatenulatus* BTL2 lipase (2W22). However, less than 40% identity was encountered with other crystal structures of *Staphylococcus hyicus* lipase (2HIH) and *Burkholderia cepacia* lipase (1OIL). As the model was built by the YASARA program, the first three crystal structures of thermostable lipases, not the mutants, were chosen as the templates to extrapolate structure of the HZ lipase. The selection was based on high sequence identity encountered from the same family of enzyme as they shared highly similar functions.

1. Zaki MJ, Bystroff C. Protein structure prediction. second ed. New York: Humana Press Inc.; 2008.

2. Sanchez R, Sali A. Comparative protein structure modeling in genomics. Journal of Computational Physics. 1999; 151: 388-401.
