# Supplementary material for: Analysis of Comparative Sequence and Genomic Data to Verify Phylogenetic Relationship and Explore a New Subfamily of Bacterial Lipases
Source: PLoS One. 2016 Mar 2;11(3):e0149851. doi: 10.1371/journal.pone.0149851 (PMC4774917; doi:10.1371/journal.pone.0149851)
Supplement: S2 Text — (DOCX) [file pone.0149851.s006.docx]

#### S2 Text. Evaluation of the predicted HZ lipase structure.

The quality evaluation of the predicted model structure could determine the information on the accuracy of 3D protein models. To verify the stereochemical quality of the model-built structure, the accuracy of parameters like bond lengths, bond angles, torsion angles and correctness of the amino acid chirality were evaluated. The predicted HZ lipase model was evaluated using three independent approaches, including PROCHECK, Verify 3D and ERRAT.

The important indicator of all stereochemical quality is the distribution of the main chain torsion angles phi, psi (Φ, Ψ) as well- determined by PROCHECK [[1](#_ENREF_1)]. The distribution of all Φ and Ψ torsion angles in a protein can be examined in a Ramachandran plot [[2](#_ENREF_2)]. The quality of the HZ lipase plot is better than the template 2DSN (T1 lipase), as 90.3% and 9.1% of residues of the crystal structure T1 lipase fell into the most-favored regions and additional allowed region, respectively. Val199 is aligned with Val203 in T1 lipase, and both were in the disallowed region. The catalytic serine residue (Ser113) lay in the generously allowed region. This criterion described by Tyndall et al. [[3](#_ENREF_3)] is a typical conformation for the “nucleophilic elbow”, which is located in a tightly constrained beta turn type structure between a β-strand and an α-helix.

Verify 3D programme evaluates the environment of each residue in a model, by its 3D profile, with respect to the expected environment as found in the high-resolution X-ray structures. Each residue of HZ lipase was assigned a structural class based on its location and environment (alpha, beta, loop, polar, nonpolar, etc.) [[4](#_ENREF_4)]. The results showed that, the average maximum score of 3D structure of HZ lipase was almost similar to the templates that used for the structure prediction. ERRAT is a protein structure verification algorithm that is especially well suited for evaluating the progress of crystallographic model building and refinement [[5](#_ENREF_5)]. Analysis by ERRAT proved the reliability of the non-bonded interaction between atoms in the HZ lipase predicted structure.

1. Laskowski RA, MacArthur MW, Moss DS, Thornton JM. PROCHECK: A program to check the stereochemical quality of protein structures. J Appl Crystallogr. 1993; 26: 283-291.

2. Ramachandran GN, Sasisekharan V. Conformation of polypeptides and proteins. Adv Protein Chem. 1968; 23: 283-438.

3. Tyndall JDA, Sinchaikul S, Fothergill-Gilmore LA, Taylor P, Walkinshaw MD. Crystal structure of a thermostable lipase from *Bacillus stearothermophilus* P1. J Mol Biol. 2002; 323: 859-869.

4. Lüthy R, Bowie JU, Eisenberg D. Assessment of protein models with three-dimensional profiles. Nature. 1992; 356: 83-85.

5. Colovos C, Yeates TO. Verification of protein structures: Patterns of nonbonded atomic interactions. Protein Sci. 1993; 2: 1511-1519.
